# Induction of Protection in Mice against a *Chlamydia muridarum* Respiratory Challenge by a Vaccine Formulated with the Major Outer Membrane Protein in Nanolipoprotein Particles

**DOI:** 10.3390/vaccines9070755

**Published:** 2021-07-07

**Authors:** Delia F. Tifrea, Wei He, Sukumar Pal, Angela C. Evans, Sean F. Gilmore, Nicholas O. Fischer, Amy Rasley, Matthew A. Coleman, Luis M. de la Maza

**Affiliations:** 1 Department of Pathology and Laboratory Medicine, University of California, Irvine, CA 92697, USA; dtifrea@uci.edu (D.F.T.); spal@uci.edu (S.P.); 2 Physical and Life Sciences Directorate, Lawrence Livermore National Laboratory, Livermore, CA 94551, USA; he4@llnl.gov (W.H.); evans98@llnl.gov (A.C.E.); gilmore24@llnl.gov (S.F.G.); fischer29@llnl.gov (N.O.F.); rasley2@llnl.gov (A.R.); coleman16@llnl.gov (M.A.C.); 3 School of Medicine, Radiation Oncology, University of California Davis, Sacramento, CA 95616, USA

**Keywords:** *Chlamydia*, vaccine, immunization, mice, nanolipoprotein particles, major outer membrane protein, intranasal challenge, telodisks, amphipols

## Abstract

*Chlamydia trachomatis* is a sexually transmitted bacterium that infects over 130 million individuals worldwide annually. To implement a vaccine, we developed a cell-free co-translational system to express the *Chlamydia muridarum* major outer membrane protein (MOMP). This approach uses a nanolipoprotein particles (tNLP) made from ApoA1 protein, amphiphilic telodendrimer and lipids that self-assemble to form 10–25 nm discs. These tNLP provide a protein-encapsulated lipid support to solubilize and fold membrane proteins. The cell-free system co-translated *MOMP* and *ApoA1* in the presence of telodendrimer mixed with lipids. The MOMP-tNLP complex was amenable to CpG and FSL-1 adjuvant addition. To investigate the ability of MOMP-tNLP+CpG+FSL-1 to induce protection against an intranasal (i.n.) *C. muridarum* challenge, female mice were vaccinated intramuscularly (i.m.) or i.n. and i.m. simultaneously 4 weeks apart. Following vaccination with MOMP-tNLP+CpG+FSL-1, mice mounted significant humoral and cell-mediated immune responses. Following the i.n. challenge, mice vaccinated with MOMP-tNLP+CpG+FSL-1 i.n. + i.m. group were protected as determined by the percentage change in body weight and by the number of *C. muridarum* inclusion forming units (IFU) recovered from the lungs. To our knowledge, this is the first time a MOMP-based vaccine formulated in tNLP has been shown to protect against *C. muridarum*.

## 1. Introduction

*Chlamydia trachomatis* is the leading bacterial sexual transmitted infection (STI) in the world, predominantly affecting young individuals [1]. In the USA it is estimated that more than 3 million new infections occur each year [2]. Multiple infections can occur during a lifetime suggesting that natural infection only induces limited protection [3]. Approximately two thirds of infected women and over 50% of infected men are asymptomatic [4,5,6,7]. In females, *C. trachomatis* acute infections can produce cervicitis and urethritis, while in males, urethritis is the most common acute clinical presentation. Acute and chronic chlamydial infections in females may lead to long-term sequelae including pelvic inflammatory disease (PID), chronic abdominal pain, ectopic pregnancy and infertility [8,9,10]. In males, epididymites and orchitis are common in young individuals but, so far there is no definitive evidence that they result infertility. Neonates born from mothers that have a genital infection may develop conjunctivitis and severe pneumonia including wheezing, cyanosis and tachypnea [10,11]. Respiratory infections have also been described in adults, particularly in those who are immunocompromised [12]. 

Attempts to control chlamydial infections using screening programs, that included antibiotics treatment, have failed [13,14]. Part of the failure of this approach may be due to arrested immunity as a result of antibiotic treatment [14]. These results underscore the need for a vaccine to prevent infection, transmission and disease [15,16,17,18,19]. Indeed, it has long been recognized that even a low efficacy vaccine could have a major impact on the epidemiology of *C. trachomatis* infections [20].

Several proteins, including the major outer membrane protein (MOMP), the chlamydial protease-like activity factor (CPAF), the polymorphic membrane proteins (Pmps) and the plasmid-encoded protein (Pgp3), have been tested as vaccine antigens in genital and respiratory mouse models using *C. muridarum* challenges [15,16,17,18]. In the genital model some of these antigens decreased vaginal shedding and inflammatory responses in the upper genital tract [21,22,23]. However, only recombinant and native MOMP have elicited protection against long-term sequelae, specifically, infertility [24,25,26,27,28,29]. 

MOMP is a 40 kDa protein that accounts for the majority of the outer membrane of chlamydial elementary bodies (EB) [30]. When isolated in its native trimeric form, MOMP can induce significant protection against infection and disease in mice [26,31]. This critical native conformation is challenging to recapitulate in recombinant MOMP (rMOMP) [32]. Previously, Olsen et al. [33,34] constructed CTH522, a Cys-free chimeric recombinant *C. trachomatis* MOMP antigen. CTH522 comprises of C. trachomatis serovar D MOMP and the variable domain 4 (VD4) from serovars D, E, F, and G. [35]. Vaccination with CTH522, adjuvanted with CAF01, induced robust humoral and cell mediated immune responses in mice and protected against vaginal shedding and inflammatory responses in the upper genital tract [33,34]. Protection against hydrosalpinx and/or infertility was not reported. This chimeric recombinant MOMP has now completed Phase 1 clinical trials [36]. However, CTH522 does not fully capture the native structure and oligomeric state of MOMP, which is shown to be of great importance for a chlamydia vaccine. In this study, we sought to address this with a novel formulation.

To optimize the structural and immunogenic properties of recombinant MOMP, He et al. [37] used an NLP comprised of a truncated ApoA1 scaffold protein and lipids that self-assemble to form discs 10–25 nm in diameter. NLPs provide a membrane-like environment that facilitate embedment of the membrane protein that is functional as well as in its native conformation. *C. muridarum* MOMP and a truncated ApoA1, Δ49ApoA1, were co-translated using T7 plasmids in the presence of lipids and mixed with telodendrimer, an amphiphilic dendrimer [38], to produce water-soluble MOMP-tNLP complexes. These studies demonstrated that this method produced MOMP multimers with similarities to native MOMP [37]. Alternatively, cell-free produced MOMP can also be solubilized by telodisks. Different from tNLPs, telodisks are nanoparticles comprised of only lipids and telodendrimers that are also disc shaped, water soluble and can support membrane proteins. With these approaches we expect that the structure of recombinant MOMP more closely will mimic its native conformation in elementary bodies (EB), the infectious form of *Chlamydia* and therefore, elicit a more robust protection.

Here, we evaluated immune responses in mice upon vaccination with co-translated MOMP-tNLP and MOMP-telodisk complexed with the Th1 adjuvant CpG-1826 and the Th2 biased adjuvant FSL-1 and investigated its ability to induce protection against an intranasal *C. muridarum* challenge. In mice, to elicit optimal protection, a subunit chlamydial vaccine, needs to induce CD4^+^ T cells with Th1-biased cytokines, specially IFN-γ, and humoral responses, in particular neutralizing antibodies [15,16,39,40,41]. CD8^+^ T cells may play a very limited role by secreting IFN-γ, but they are not cytotoxic [15,40]. Here, by including in the formulation adjuvants combinations that elicit both Th1 and Th2 responses, we predicted that protective cellular and humoral responses will be induced.

## 2. Materials and Methods

### 2.1. C. muridarum Stocks

The *C. muridarum* strain NiggII (previously called *C. trachomatis* mouse pneumonitis (MoPn) biovar; American Type Culture Collection, Rockville, MD, USA) was grown in HeLa-229 cells (American Type Culture Collection, Rockville, MD, USA) using Eagle’s minimal essential medium supplemented with 5% fetal calf serum [42]. Elementary bodies (EB) were purified as previously described and stored in sugar phosphate glutamate buffer (SPG) at −80 °C [30]. The number of *C. muridarum* inclusion forming units (IFU) was determined in HeLa-229 cells and the inclusions were stained with a pool of monoclonal antibodies generated in our laboratory [26].

### 2.2. Cell-Free Preparation of MOMP-tNLP

MOMP-tNLPs were prepared using cell-free methods previously described [37]. Briefly, plasmids encoding the codon optimized *C. muridarum* MOMP gene and His-tagged scaffold Δ49ApoA1 gene were added to a 1 mL RTS 500 ProteoMaster *Escherichia coli* HY cell-free reaction mixture (Biotechrabbit GmbH, Hannover, Germany) containing 1,2-dimyristoyl-sn-glycero-3-phosphocholine (DMPC) (Avanti Polar Lipids, Alabaster, AL, USA) and telodendrimer PEG^5k^-CA_8_. The telodendrimer is a polymer of polyethylene glycol complexed with branched cholate molecules [38]. The reactions were incubated at 30 °C for 14–18 h. in a floor shaker at 300 rpm. The MOMP-tNLP complex was purified by immobilized nickel affinity chromatography using cOmplete His-Tag Purification Resin (Roche Molecular Diagnostics, Basel, Switzerland). Elutions containing MOMP-tNLP were pooled and dialyzed against PBS (pH 7.4). Purified MOMP-tNLP were analyzed by SDS-PAGE densitometry for purity and degree of MOMP insertion. Total protein concentration was measured using Qubit (Invitrogen, Carlsbad, CA, USA). To verify molecular weight and concentration of the cell-free produced MOMP, samples were compared to purified rMOMP with known concentration through SDS-PAGE. For a negative control, empty tNLPs were prepared using the same procedure as that of MOMP-tNLP, except no MOMP encoding DNA was added to the cell-free reaction. Endotoxin levels were checked using the Endosafe^®^-PTS™ (Charles River, Charleston, SC, USA) endotoxin testing system based on Limulus Amebocyte Lysate (LAL) assay. The average endotoxin level of the MOMP-tNLPs formulations was 7.80 ± 4.87 EU/µg protein while the average endotoxin level of empty tNLPs was 0.58 ± 0.14 EU/µg protein.

### 2.3. Cell-Free Preparation of MOMP-Telodisk

MOMP-telodisks were prepared using cell-free methods similar to the preparation of MOMP-tNLP. Briefly, MOMP plasmid was added to a 1 mL RTS 500 ProteoMaster *E. coli* HY cell-free reaction mixture containing DMPC and His-tagged telodendrimer PEG^5k^-CA_8_-His. The reactions were incubated at 30 °C for 14–18 h. in a floor shaker with shaking at 300 rpm. The MOMP-telodisk complex was purified by immobilized nickel affinity chromatography. Elutions containing MOMP-telodisks were pooled and further purified by size exclusion chromatography (SEC) using a Superdex 200 10/300 column (GE Healthcare, Piscataway, NJ, USA) run with PBS buffer +0.25%PEG_2000_. Addition of 0.25% PEG_2000_ in PBS was important for effective elution of MOMP-telodisk. Purified MOMP-telodisk concentration was measured using Qubit (Life Technologies, Carlsbad, CA, USA)). Empty telodisks were prepared using the same procedure except without MOMP encoding DNA. Endotoxin levels were checked as above. The average endotoxin level of the MOMP-telodisks formulations was 1.00 ± 0.76 EU/µg protein.

### 2.4. Western Blot and Dot Blot Analyses

Western blotting and dot blotting were performed using PVDF membranes (MilliporeSigma, Burlington, MA, USA). For Western blotting, samples were resolved with SDS-PAGE. The gels were transferred using the iBlot system (Life Technologies, Carlsbad, CA, USA) according to manufacturer’s instructions. Blots were incubated overnight at 4 °C in Odyssey Blocking Buffer (LI-COR Biotechnology, Lincoln, NE, USA) containing 0.2% Tween 20 and either 0.5 mg/mL mAb40 [monoclonal antibody to the variable domain 1 (VD1) of MOMP] or 0.2 mg/mL Penta-His antibody (Qiagen, Hilden, Germany) directed against the ApoA1 His-tag, diluted 1:1000 for mAb40 and 1:500–1:1000 for Penta-His. Blots were washed 3 times for 5 min with PBS-T (50 mm NaH_2_PO_4_, 300 mm NaCl, 0.2% Tween 20, pH 7.4) and incubated for 1 h in blocking buffer containing 1 mg/mL IRDye 800CW goat (polyclonal) anti-mouse IgG (heavy and light) (LI-COR Biosciences, Lincoln, NE, USA. 1:10,000). Blots were washed again with PBS-T and imaged with a LI-COR Fc imager at 800 nm. For dot blots, 3 μg of purified MOMP-tNLP and empty tNLP were blotted using the Bio-Dot apparatus (1706545, Bio-Rad, Hercules, CA, USA) according to the manufacturer’s instructions. Blots were developed using the same methods described for Western blotting. 

### 2.5. Adjuvant Addition 

CpG ODN-1826 (Biosearch Technologies, Novato, CA, USA) was custom synthesized with a cholesterol moiety at the 5′ end (referred herein as CpG). The insertion of CpG into NLPs or telodisks is mediated by hydrophobic interactions and has been demonstrated in a previous study [43]. 

To assess whether the vaccine adjuvant FSL-1 (InvivoGen, San Diego, CA, USA) could be loaded into NLPs, NLPs containing 35% DGS-NTA(Ni) lipid and 65% DOPC using recombinantly expressed mouse ApoE4 were prepared as previously described [43,44]. Concentration of the purified particles was obtained by measuring the absorbance at 280 nm using a Nanodrop, and assumes a particle content of 5 scaffold proteins [45]. FSL-1 in water was then added to the NLP solution at the specified FSL-1:NLP ratio and allowed to mix for 30 min. FSL-1:NLP solutions were then analyzed by size exclusion chromatography (SEC) using a Superdex 200 10/300 column (GE Healthcare, Piscataway, NJ, USA) while measuring absorbance of the particle solutions at 214 nm. 

During the vaccine preparation, both immune-stimulatory adjuvants CpG and FSL-1 were added directly to the purified MOMP-tNLP and empty tNLP at the specified concentration for each group. The adjuvanted samples were analyzed by analytical SEC using Superdex 200, 5/150 GL column, (GE Healthcare, Piscataway, NJ, USA) in PBS buffer (0.5 mL/m in flow rate). The adjuvanted MOMP-tNLP and empty tNLP were stored at 4 °C prior to animal use.

### 2.6. Purification and Formulation of C. muridarum rMOMP in Amphipols (MOMP/APol)

The *C. muridarum* rMOMP was extracted from the *E. coli* inclusion bodies as described by Marston [46]. The pellet of the rMOMP was solubilized in TEN buffer with 8 M urea, 0.1 mM PMSF and 0.02 mM DTT to a concentration of 10 mg/mL. Following solubilization the rMOMP was loaded onto a Sephacryl-S-300 column (1 × 50 cm; Sigma-Aldrich, St. Louis, MO, USA), which was pre-equilibrated with 100 mM Tris-HCl, pH 8.0, 200 mM NaCl, 10 mM EDTA, 0.2 mM DTT, and 0.05% Z3-14 (Anatrace; Maumee, OH, USA) and the peak fractions were pooled [32]. The apparent MW and purity of *C. muridarum* rMOMP was determined by 10% tricine-SDS-PAGE. Using the limulus amoebocyte assay (BioWhittaker, Inc., Walkersville, MD, USA), the rMOMP was found to have less than 0.05 EU of LPS/mg of protein. The rMOMP was transferred from zwittergent Z3-14 to amphipols (APols) A8-35 (Anatrace, Maumee, OH, USA) by incubation at room temperature for 2 h at a weight ratio of 1/4 [27,31]. The Z3-14 detergent was removed with rehydrated BioBeads SM-2 Adsorbent (Bio-Rad, Hercules, CA, USA) at a weight ratio of 1/2.5 (Z3-14/Biobeads) by incubation for 16 h at 4 °C, after which the beads were separated by centrifugation [27,31].

### 2.7. Pilot Experiment

Five-week-old female BALB/c (H-2^d^) mice were purchased from Charles River Laboratories (Hollister, CA, USA). All mice were maintained at the University of California, Irvine Vivarium and experiments carried out in accordance with the IACUC guidelines.

To evaluate the safety, reactogenicity and protective ability of MOMP-tNLP and MOMP-telodisk preparations, a pilot study was performed with six groups of mice, seven animals per group. The two experimental groups were: (1) MOMP (10 μg/mouse/immunization) in Telodisks adjuvanted with CpG (1 μg/mouse/immunization) and (2) MOMP (10 μg/mouse/immunization) in tNLP adjuvanted with CpG (1 μg/mouse/immunization). Mice were immunized twice by the intranasal (i.n.), 20 μL/nostril, plus intramuscular (i.m.), 50 μL/hind leg, routes at 4-week intervals. The positive control was vaccinated once i.n. with 10^4^ inclusion forming units (IFU) of *C. muridarum* in 20 μL of Eagle’s Minimum Essential Medium (EMEM). Two antigen controls and one vaccine negative controls were immunized with: (1) Telodisks plus CpG (1 μg/mouse/immunization); (2) tNLP plus CpG (1 μg/mouse/immunization); and (3) PBS with 0.25% PEG_2000_. The negative controls were immunized using the same protocol as the experimental groups.

Six weeks after the last immunization mice were challenged i.n. with 10^4^ *C. muridarum* IFU. The body weight was determined before the challenge and then daily for 10 consecutive days at which time the animals were euthanized, their lungs harvested, weighed, and the number of *C. muridarum* IFU determined.

### 2.8. Mouse Immunizations

To assess the effectiveness of MOMP-tNLP adjuvanted with CpG and FSL-1, BALB/c mice were vaccinated twice four weeks apart with 10 µg of *C. muridarum* MOMP/mouse/immunization in tNLP, containing 1 ug of CpG and 1 ug of FSL-1 per mouse/immunization. A group of mice was vaccinated simultaneously by the i.n., 20 μL/nostril, plus i.m., 50 μL, routes while another group was immunized only i.m., 50 μL. A positive control received rMOMP 10 μg/mouse/immunization, produced in *E. coli* and adjuvanted with 10 μg/mouse/immunization of CpG-1826 (Tri-Link, San Diego, CA, USA) plus Montanide ISA 720 VG (SEPPIC Inc., Fairfield, NJ, USA; 70% of total vaccine volume) [32]. Montanide ISA 720 VG was delivered only i.m. The second positive control was immunized once i.n. with 10^4^ IFU of *C. muridarum* EB in 20 μL of EMEM. Two negative controls were included: one received empty tNLP with adjuvants twice i.n. + i.m. four weeks apart and the other one was inoculated i.n. + i.m. once with 20 μL of PBS (pH 7.2). The experiments were replicated once.

### 2.9. ELISA Antibody Titers

Blood was collected the day before the i.n. challenge and *C. muridarum* specific antibody titers in serum were determined by an ELISA as described [26]. Pre-immunization sera were used as negative controls. In brief, 96-well plates were coated with 100 µL of *C. muridarum* EB at a concentration of 10 µg of protein/mL. In addition, the antibody titers were also determined utilizing MOMP-tNLP as the antigen (0.1 μg/well). Serum was added to each well in 2-fold serial dilutions. After incubation, the plates were washed and incubated with horseradish peroxidase-conjugated goat anti-mouse IgG antibodies (BD Pharmingen, San Diego, CA, USA). The binding was measured in an EIA reader (Labsystem Multiscan, Helsinki, Finland). The geometric mean titers (GMT) are expressed as the reciprocal of the dilution.

### 2.10. In Vitro Neutralization Titers

In vitro neutralization assays were performed as published [47]. *C. muridarum* (1 × 10^4^ IFU) were added to mice sera and two-fold serial dilutions were made with Ca^2+^ and Mg^2+^ free PBS, pH 7.2, supplemented with 5% guinea pig serum. After incubation for 45 min at 37 °C, the mixtures were inoculated by centrifugation into HeLa-229 cells grown on shell vials. Following 30 h of incubation at 37 °C the monolayers were fixed and stained with a pool of mAbs [48]. The titer of a sample was the dilution that yielded 50% neutralization relative to the negative control serum from PBS immunized mice.

### 2.11. Cellular Immune Responses

A T-cell lymphoproliferative assay (LPA) was performed as described [26,31]. Briefly, spleens from mice were collected, teased and enriched for T-cells by passage over a nylon wool column. T-enriched cells (>95%) were counted and 10^5^ cells/well were aliquoted into a 96-well plate. Antigen presenting cells (APC) were prepared by irradiating splenocytes with 3300 rads. UV inactivated *C. muridarum* EB were added at a concentration of 5 EB to 1 APC. MOMP/APol was used at a concentration of 1 μg/well. As negative control wells received medium alone and as positive control concanavalin A (5 μg/mL). T-cells were stimulated for 48h and levels of IFN-γ and IL-6 were determined in supernatants using commercial kits (BD Pharmingen, San Diego, CA, USA) [49].

### 2.12. Evaluation of the Infection Following the i.n. Challenge

Four weeks after the last immunization anesthetized mice were challenged i.n. with 10^4^ IFU of *C. muridarum* [32,50]. After the i.n. challenge, mice were weighed for 10 days [32]. At day 10 post-challenge (D10 p.c.) mice were euthanized, their lungs weighed, homogenized and serial 10-fold dilutions were used to infect HeLa-229 cells. Following centrifugation, the plates were incubated for 30 h at 37 °C in a 5% CO_2_ incubator. Inclusions were stained with a pool of *C. muridarum*-specific mAbs and were counted [48] using light microscopy. The limit of detection (BLD) was: <50 *C. muridarum* IFU/lungs/mouse.

To determine the local humoral and cellular immune responses in the lungs, the titers of *C. muridarum*-specific IgA and levels of IFN-γ in lung’s supernatants at D10 p.c. were determined by an ELISA as described [51].

### 2.13. Statistical Analyses

Student’s *t*-test was performed to compare antibody ELISA titers and neutralization titers. The Mann–Whitney U test was employed to compare levels of cytokines in spleen supernatants. The Kruskal–Wallis with Dunn’s multiple comparisons test was used to compare the number of *C. muridarum* IFU in the lungs, and the one-way ANOVA with Tukey’s multiple comparisons test was performed to compare the lungs weight, body weight, and levels of cytokines in the lungs. Repeated measures ANOVA analyses were conducted to compare changes in mean body weight over the 10 days of observation.

## 3. Results

### 3.1. Expression, Characterization of MOMP-tNLP and MOMP-Telodisk

**Figure 1:**
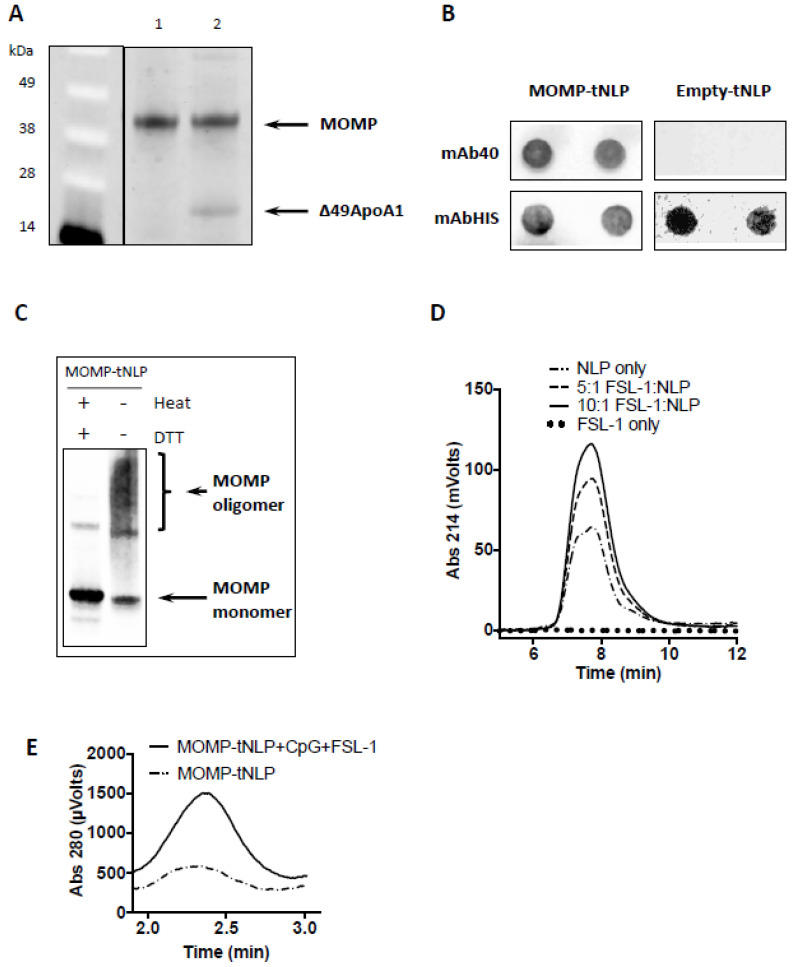
Expression, characterization and adjuvant addition of MOMP-tNLP. (**A**) SDS-PAGE of purified MOMP-tNLP. Left lane (MW markers) Lane 1: One µg of purified recombinant MOMP. Lane 2: Purified MOMP-tNLP containing one µg of MOMP. The gel densitometry values are: lane 1 top band, 15,971.3; lane 2 top band 15,887.4; lane 2 bottom band 3629.0 (Ana-lyzed by ImageJ). (**B**) Dot blot of purified MOMP-tNLP and empty tNLP probed with anti-MOMP mAb40, and anti-Histag antibody mAbHIS. Blotting is done in duplicate. Histag is on the N-terminal of tNLP scaffold Δ49ApoA1. (**C**) MOMP forms higher order structures in MOMP-tNLPs as analyzed by Western blot. In the right lane, the presence of higher order bands indicates MOMP multimer conformation. In the left lane, heat and denaturant break down higher order MOMP structures (samples were boiled for 5 min in the presence of 50 mM DTT). The gel densitometry values are: left lane top bands, 841.9; left lane bottom band, 9239.1; right lane top bands 6077.9; right lane bottom band 3643.4 (Analyzed by ImageJ). (**D**) FSL-1 adjuvant incorporation into NLPs. FSL-1 is a lipopeptide that can be anchored in the NLP bilayer via the acyl chains. SEC demonstrates dose-dependent incorporation of FSL-1, evidenced by an increase in absorbance at 214 nm. (**E**) SEC traces of MOMP–tNLP particles with and without adjuvant addition. Same amount of MOMP-tNLP were loaded onto SEC. The increase in A280 indicates association of adjuvant CpG with the MOMP-tNLP.

For MOMP-tNLP, the cell-free co-translation reaction resulted in expression of both MOMP and Δ49ApoA1, which formed a de novo-soluble complex that was purified through Ni affinity purification. The MOMP concentration was confirmed by direct comparison to purified rMOMP of known concentration via gel densitometry (Figure 1A). The average total affinity purified yield was 1.5 mg MOMP-tNLP complex per mL reaction. Dot blots of purified MOMP-tNLP and empty tNLP probed with mAb-His and mAb40 showed proper incorporation of MOMP in the tNLPs (Figure 1B). In addition, the purified MOMP within the tNLPs formed higher order oligomers, as demonstrated by Western blot (Figure 1C). In the absence of both heat and reducing agent, bands corresponding to higher order structures were observed (Figure 1C, right lane). These disappeared when samples were heated in the presence of DTT (Figure 1C, left lane).

For MOMP-telodisk, it is also purified by Ni affinity purification after cell-free reactions (Supplementary Figure S1A). Dot blots of purified MOMP-telodisks probed with mAb-His and mAb40 confirmed incorporation of MOMP in the telodisks (Supplementary Figure S1B). The purified MOMP-telodisk also showed higher order oligomers in Western blot in the absence of heat and reducing agent (Supplementary Figure S1C).

### 3.2. Adjuvant Addition

NLPs are known to be excellent carriers for immune adjuvant molecules such as CpG, whereby the cholesterol tag anchors the adjuvant in the NLP bilayer through hydrophobic interactions [43]. This same strategy was used to incorporate the amphipathic lipopeptide FSL-1 into the NLP. NLPs were mixed and incubated with FSL-1 at increasing molar ratios and analyzed by SEC. The adjuvanted NLP complex showed a dose dependent increase in 214 nm (used to monitor absorption of peptide bonds), indicating successful incorporation of FSL-1 (Figure 1D).

MOMP-tNLP was characterized by SEC after incubation with CpG and FSL-1. The adjuvanted MOMP-tNLP showed a similar retention time compared with the non-adjuvanted MOMP-tNLP. However, the adjuvanted MOMP-tNLP showed a much higher absorption at 280 nm, indicating successful loading of dual adjuvant molecules. The increase in the signal intensity at 280 nm of the MOMP-tNLP peak is indicative of the binding of CpG, which has a high molar absorptivity (Figure 1E) [43].

### 3.3. Pilot Experiment

Six groups of mice were vaccinated and challenged i.n. with *C. muridarum*. The MOMP-tNLP+CpG and the MOMP-telodisk+CpG groups each had its own negative control group without MOMP. In addition, a negative vaccine control group received PBS+0.025%PEG_2000_ and a positive control group was immunized with viable *C. muridarum* EB.

After immunization, mice were assessed daily for the appearance of ruffled fur, behavioral changes, decreased mobility or loss of body weight. Mice showed mild signs of distress for 4–5 days and then recovered.

Following the i.n. challenge, positive control mice immunized i.n. with *C. muridarum* viable EB maintained their body weight over the 10 days of the experiment (Supplementary Figure S2). In contrast, the three negative control groups at day 3 post-challenge (p.c.) rapidly started to lose body weight that continued for the 10 days of the experiment. Over the 10-day period, the experimental group vaccinated with MOMP-telodisk+CpG lost body weight similar to the negative controls. Mice vaccinated with MOMP-tNLP+CpG lost less weight over the 10-day period than the controls immunized with tNLP+CpG, or the group receiving PBS with 0.25% PEG_2000_ (*p* < 0.05).

By D10 p.c. mice vaccinated with MOMP+tNLP+CpG had lost significantly less body weight (10%) than the negative control group (20%) (*p* < 0.05) (Supplementary Figure S3A). Mice vaccinated with MOMP in Telodisks were not protected when compared with the negative control (*p* > 0.05).

To evaluate the local inflammatory responses elicited by the *C. muridarum* i.n. challenge, lungs were collected and weighed at D10 p.c. (Supplementary Figure S3B). Typically, unprotected mice have shown increased lungs weight after challenge. The groups vaccinated with MOMP+tNLP+CpG had lighter lungs (0.28 gr), than the negative control immunized with tNLP+CpG (0.31 gr) (*p* < 0.05). Mice vaccinated with MOMP-telodisk+CpG (0.28 gr) were not protected when compared with the telodisk+CpG control (0.30 gr) (*p* > 0.05).

Protection against infection was determined by quantifying the number of *C. muridarum* IFUs recovered from the lungs at D10 p.c. (Supplementary Figure S3C). Mice vaccinated with MOMP+CpG+tNLP had significantly less *C. muridarum* IFU in their lungs (median, 12 × 10^6^) when compared with the control inoculated with CpG+tNLP (170 × 10^6^) (*p* < 0.05). The group vaccinated with MOMP-telodisks-CpG had similar number of IFU (101 × 10^6^) when compared with its respective negative control immunized with CpG+Telodisks (159 × 10^6^) (*p* > 0.05).

The pilot study showed that mice immunized with MOMP-tNLP were better protected from challenge than those immunized with MOMP-telodisks.

### 3.4. Characterization of the Humoral Immune Responses Following Vaccination

**Figure 2:**
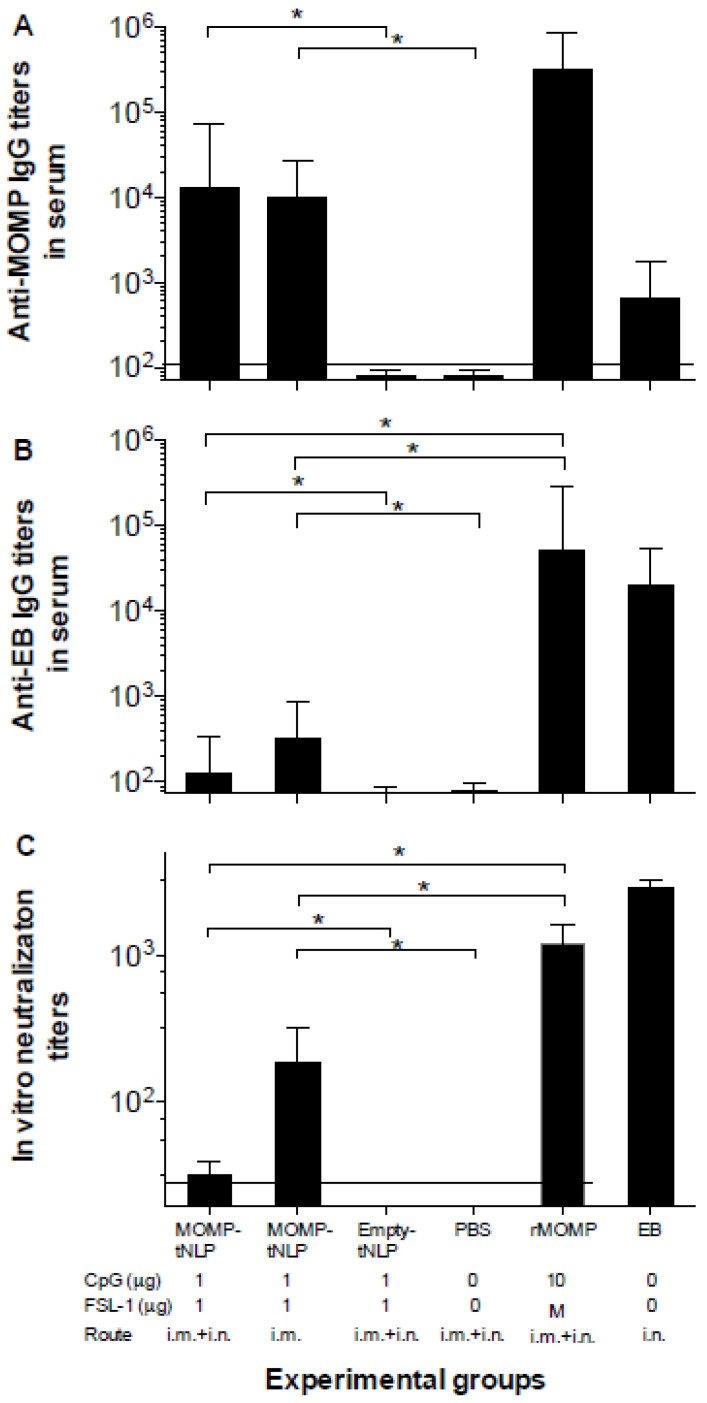
**Serum antibody titers.** (**A**) Anti-*C. muridarum* MOMP and (**B**) anti-*C. muridarum* EB IgG antibody titers. (**C**) In vitro neutralizing *C. muridarum* EB titers. Serum samples were collected before immunization as background control, and antibody titers were determined the day before the i.n. challenge. Horizontal dotted lines represent the limit of detection. * *p* < 0.05 by ANOVA.

Based on the pilot study results, we decided to proceed with MOMP-tNLP to further assess the immune response of the formulation with both CpG and FSL-1 adjuvants. Background control sera were collected prior to immunization. To evaluate humoral immune responses elicited by vaccination, sera were collected prior to challenge. Total IgG levels were quantified using *C. muridarum*-EB and MOMP-tNLP as antigens. Significantly higher specific IgG GMT were observed when MOMP-tNLP was used as the antigen (Figure 2A). In mice immunized with MOMP-tNLP+CpG+FSL-1 by the i.m. + i.n. routes the MOMP-tNLP specific IgG GMT was 12,800 while for mice immunized by the i.m. route only, the MOMP-tNLP specific IgG GMT was 10,159. The IgG GMT in the positive controls immunized with rMOMP, or EB were 325,100 and 635, respectively. As shown in Figure 2B, levels of antibodies to EB were low in mice immunized with MOMP-tNLP. The highest IgG GMT, 317, was observed in mice vaccinated with MOMP-tNLP+CpG+FSL-1 using the i.m. route only. The GMT in the control groups immunized i.n. with rMOMP or EBs were 51,200 and 25,600, respectively. The negative controls immunized with tNLP+CpG+FSL-1 and the PBS group had IgG titers below the level of detection.

The in vitro neutralization titers in mice immunized with MOMP-tNLP+CpG+FSL-1 i.m. + i.n. was 32 and in the group vaccinated only i.m. was 185 (Figure 2C). Mice vaccinated with live EB had a GMT of 2880 and for those immunized with rMOMP it was 1131. Together, these data showed that MOMP-tNLPs induced robust humoral response.

### 3.5. Assessment of Cell Mediated Immune (CMI) Responses Following Vaccination

**Figure 3:**
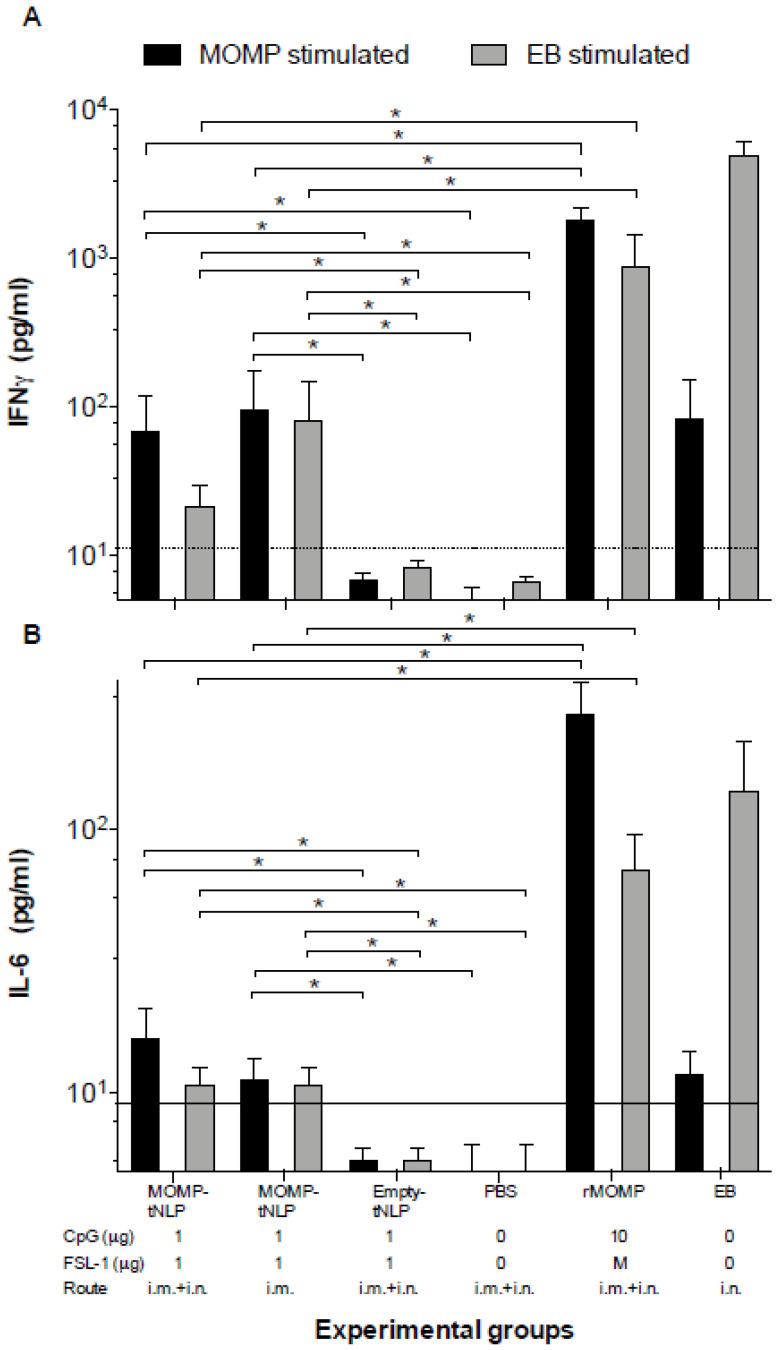
Levels of (**A**) IFN-γ and (**B**) IL-6 produced by T cells stimulated with EB or MOMP/APols the day before the *C. muridarum* i.n. challenge. The day before the challenge, mice were euthanized, their spleens collected, and T-cells purified using a nylon wool column. Purified T-cells were stimulated in vitro with *C. muridarum*-EB or with -rMOMP in APols. Cytokines were measure in the tissue culture supernatants. Horizontal dotted line represents the limit of detection. * *p* < 0.05 by ANOVA.

To determine CMI responses elicited by vaccination, four mice from each group were euthanized 24 h prior to the i.n. challenge. T-cells were collected from the spleen, processed into a single cell suspension and cultured in the presence of *C. muridarum*-EB or rMOMP. After 48 h, culture supernatants were harvested and levels of IFN-γ and IL-6, as markers of Th1 and Th2 responses, respectively, were quantified using standard ELISA assays (Figure 3). Levels of IFN-γ (pg/mL) in T-cell supernatants from mice immunized with MOMP-tNLP+CpG+FSL-1 i.m. + i.n. and restimulated with EB were significantly higher than the control groups either immunized with empty tNLP or PBS while both levels were significantly lower than rMOMP or EB vaccinated animals. Levels of IL-6 (pg/mL) were similar for both MOMP-tNLP+CpG+FSL-1 immunized groups (i.m. and i.n. + i.m.). and were significantly lower than the rMOMP or EB vaccinated mice. To conclude, levels of IFN-γ and IL-6, although significant in mice immunized with MOMP-tNLP+CpG+FSL-1, were lower than those elicited in mice vaccinated with rMOMP or EB.

### 3.6. Changes in Body Weight Following the C. muridarum i.n. Challenge

To determine the ability of the MOMP-tNLP+CpG+FSL-1 formulations to elicit protective immune responses, mice were i.n. challenged with *C. muridarum*. To assess the systemic effects of the challenge, the body weight of each mouse was determined for 10 consecutive days.

**Figure 4:**
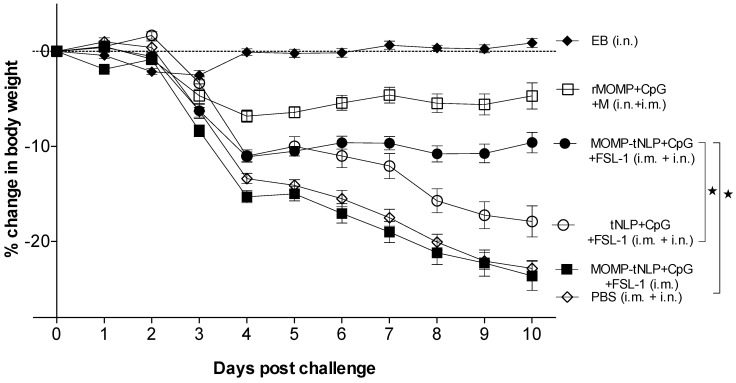
Daily percentage change in mean body weight following the i.n. challenge. * *p* < 0.05 by the Repeated Measures 2-way ANOVA.

As shown in Figure 4, the positive control immunized i.n. with *C. muridarum* EB lost ~3% of their initial mean body weight by day 4 (D4) after which animals recovered their initial body weight. The group vaccinated with rMOMP lost body weight until D4 and then maintained the weight. Mice immunized with MOMP-tNLP+CpG+FSL-1 i.m. + i.n. lost ~12% of their initial body weight by D4 and then maintained their body weight until D10. In contrast, animals that received tNLP+CpG+FSL-1 continuously lost weight over the 10-day time course. Control animals inoculated with PBS lost the most weight during the 10 days of monitoring similar to mice immunized only i.m. with MOMP-tNLP+CpG+FSL-1.

**Figure 5:**
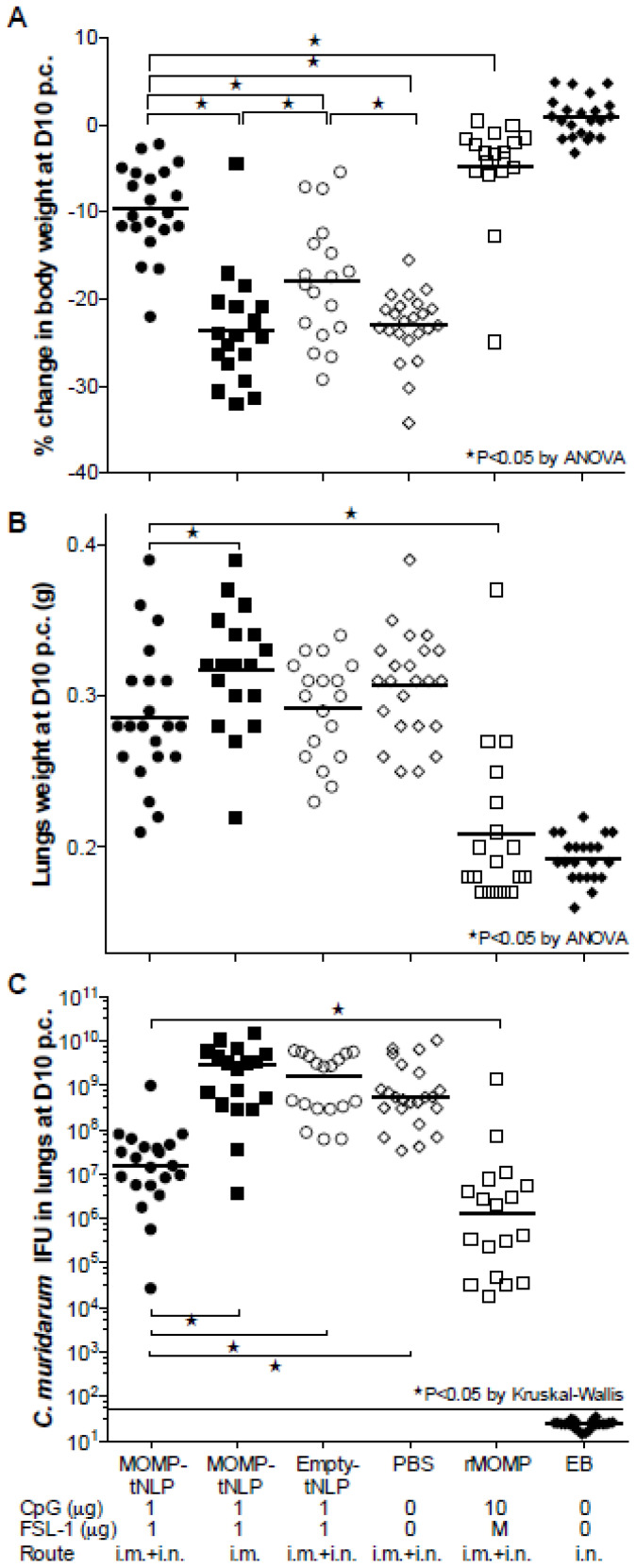
Systemic and local disease burden following the i.n. challenge with *C. muridarum*. (**A**) Percentage change in mean body weight at D10 following the i.n. challenge. The mean is shown as a horizontal line. Each symbol represents a single animal. (**B**) Lungs weight (g) at D10 after the i.n. challenge. The mean is shown as a horizontal line. Each symbol represents a single animal. (**C**) Number of *C. muridarum* IFU recovered from the lungs at D10 after the i.n. challenge. The median is shown as a horizontal line. Each symbol represents a single animal. * *p* < 0.05.

Based on body weight changes at D10 post-challenge (p.c.) mice vaccinated with MOMP-tNLP+CpG+FSL-1 via the i.m. + i.n. routes (−9.60%; *p* < 0.05) exhibited significant protection when compared to the control tNLP+CpG+FSL-1 immunized animals (−17.90%) (Figure 5A). However, the group immunized only i.m. was not protected (−23.63%; *p* > 0.05) compared to the PBS immunized group. The group vaccinated with rMOMP lost 5% of their initial body weight while the PBS group lost 23.03%. Mice immunized with EB regained their original body weight.

### 3.7. Lungs’ Weight

Mice vaccinated with MOMP-tNLP+CpG+FSL-1, by i.m. + i.n. routes, had statistically lower lungs weight compared to the group immunized by the i.m. route only. Nevertheless, there was no statistical difference in the lungs weight between any of the groups when compared with negative controls immunized with tNLP+CpG+FSL-1 or PBS (*p* > 0.05) (Figure 5B). Lungs weights in mice immunized with rMOMP- or EB-indicated robust protection.

### 3.8. Number of C. muridarum IFU Recovered from the Lungs

Based on the number of IFU recovered (median 15.7 × 10^6^), mice immunized i.m. + i.n. with MOMP-tNLP+CpG+FSL-1 were the best protected. This number was significantly lower when compared with mice vaccinated only i.m. (2843 × 10^6^) and the negative control immunized with tNLP+CpG+FSL-1 (1560 × 10^6^) (*p* < 0.05). The group of mice vaccinated with rMOMP had 1.2 × 10^6^ IFU. No IFU were recovered from the positive control immunized i.n. with EB (limit of detection < 50 IFU/mouse/lungs) (Figure 5C).

### 3.9. Local Immune Responses in the Lungs at D10 p.c.

**Figure 6:**
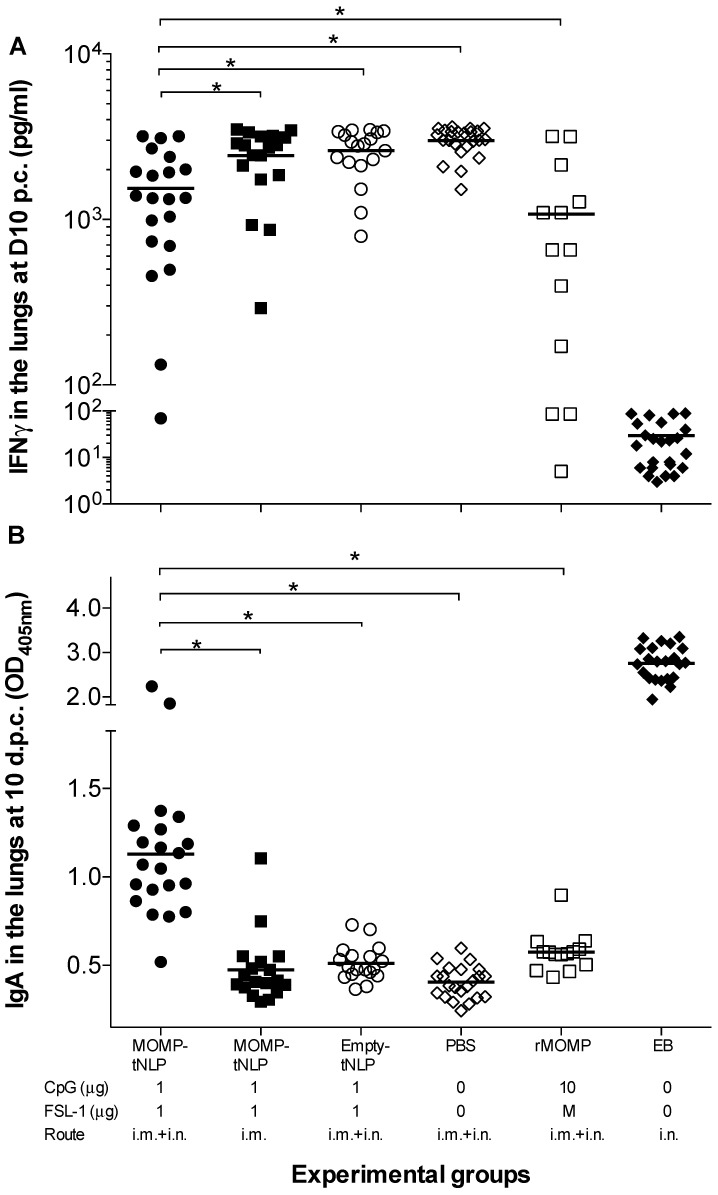
Levels of IFN-γ and titers of *C. muridarum*-specific IgA in the lungs at D10 p.c. (**A**) Levels of IFN-γ (pg/mL) detected in lung supernatants at D10 after the i.n. challenge. The mean is shown as a horizontal line. Each symbol represents a single animal. (**B**) Levels of *C. muridarum*-specific IgA (OD_405_) detected in lung supernatants at D10 following i.n. challenge. The mean is shown as a horizontal line. Each symbol represents a single animal. * *p* < 0.05.

As an additional parameter to evaluate the local control of the infection, levels of IFN-γ were determined in lung supernatants (Figure 6A). Of the groups vaccinated with MOMP-tNLP+CpG+FSL-1, the lowest levels of IFN-γ (1538; mean pg/mL) were observed in mice immunized by a combination of i.m. + i.n. routes, significantly lower than the PBS control (3002; *p* < 0.05). Very low levels of IFN-γ were observed in the positive control immunized with EB (32), indicative of local control of the infection, while high levels of IFN-γ were detected in the groups vaccinated with MOMP-tNLP+CpG+FSL-1 by the i.m. route only and the negative controls immunized with tNLP+CpG+FSL-1 or PBS (2431, 2613 and 3002, respectively). The level of IFN-γ in mice vaccinated with rMOMP was 1077; *p* < 0.05).

As a parameter of the local memory immune responses elicited by vaccination, levels of specific *C. muridarum* IgA were quantified in lungs supernatants (Figure 6B). Mice vaccinated with MOMP-tNLP+CpG+FSL-1 by the i.m. + i.n. routes had higher IgA levels (1.130; mean OD_405_) than those vaccinated only i.m. (0.475) (*p* < 0.05). Mice vaccinated with rMOMP had an IgA level of 0.575. The highest levels of IgA were observed in control mice immunized i.n. with live EBs (2.763) while the lowest level was determined in the negative PBS control (0.405).

## 4. Discussion

The number of *C. trachomatis* infections continues to increase, and there is an urgent need to implement a vaccine. Here, NLPs were used to support and deliver recombinant *C. muridarum* MOMP by mucosal and systemic routes using CpG and FSL-1 as adjuvants. The cell-free approach allowed for production, purification, and adjuvant addition within a single day to rapidly produce formulations for screening. Some of the vaccine formulations elicited significant humoral and cellular immune responses to *C. muridarum* EB and rMOMP. Mice were subsequently challenged in the nostrils and, based on changes in body weight and number of *C. muridarum* IFU recovered from the lungs, mice were partially protected. Specifically, when mice were simultaneously immunized through i.m. and i.n. routes, the MOMP tNLP vaccine elicited protection, highlighting the importance of the choice of routes. To our knowledge, this is the first time that *C. muridarum* MOMP formulated in a bio-mimetic nanoparticle has been shown to elicit significant protection against infection and disease.

MOMP accounts for 60% of the outer membrane mass of *Chlamydia* elementary bodies and is highly antigenic [52]. B-cell epitopes have been mapped to all four variable domains (VDs) of MOMP while T-cell epitopes have been mainly identified in the constant domains (CDs), although some of them were also located in the VDs [35,53,54]. Native MOMP can be generated in small quantities, but escalating production to vaccinate humans will be technically difficult and costly. Recombinant MOMP, although not as efficacious at eliciting protection as native MOMP, can easily be produced in *E. coli*. However, since MOMP is an intrinsic membrane protein, it requires detergents or amphipols to keep it soluble in an aqueous solution [25,31]. The presence of eight cysteine residues in MOMP also creates challenges to obtain an antigen that has consistent structural and antigenic properties.

To evaluate the safety, reactogenicity, and protective ability of tNLP- and telodisk- MOMP preparations, a pilot study was performed. Except for the group inoculated with PBS all mice lost body weight following the i.n. plus i.m. immunization. By 4-5 days post-immunization, the animals had recovered their initial body weight and behaved normally. No significant differences between the five groups were observed.

Following challenge, as determined by changes in body weight, lung weight and number of *C. muridarum* IFU recovered from the lungs, mice vaccinated in MOMP-tNLP were better protected than those immunized with MOMP-telodisks. tNLP differs from telodisk by the presence of scaffold proteins which corral the disk shape membrane and further stabilize as well as solubilize the embedded membrane protein [38]. The advantage of tNLP over telodisk is likely due to the increased solubilization and stbility that the tNLP scaffold protein provided to maintain the native state MOMP. Based on these results, we decided to proceed using MOMP-tNLP rather than MOMP-telodisks for the definitive experiments.

The tNLPs were used to keep MOMP soluble and to fold it under conditions that mimic the lipid bilayer of a membrane. Previous results showed that the tNLP supporting scaffold could solubilize MOMP in an oligomeric form as a functional porin [37]. Furthermore, the proper insertion of MOMP into the NLP lipid bilayer should allow for the maximum exposure of the MOMP extracellular domains, including all of the antigenic VDs. An additional benefit of NLP is that they can be used to incorporate a variety of adjuvants for screening vaccine formulations. Using highly purified proteins as vaccine antigens requires the addition of adjuvants to boost local innate and adaptive immune responses [55]. Moreover, co-delivery of adjuvants along with the antigen increases the efficacy of vaccines [56,57]. There is evidence from both human and animal studies that cellular immunity, with Th1 responses and IFN-y production, is needed to recover from infection. In the mouse model, CD4 T cells are required for protection against a *C. muridarum* challenge, while CD8 T cells do not seem to play a major role in protection [58]. IFN-y was likely produced by CD4^+^ T cells, and not by CD8^+^ T cells, since they are predominant in the female genital tract [59]. In addition, during re-infection, antibodies control the initial bacterial load and aid in resolution of the infection [40,58]. Furthermore, in humans, an inverse correlation has been found between shedding of *C. trachomatis* and immunoglobulin A (IgA) levels in cervical secretions [60]. Thus, to formulate the MOMP-tNLP vaccine we used a combination of CpG, a TLR-9 agonist that elicits Th1 immune responses, and FSL-1, a TLR-2 agonist that induces Th2-biased responses.

Here, mice that were vaccinated using a combination of a mucosal (i.n.) and systemic (i.m.) routes were better protected than the animals immunized only i.m. The utilization of a mucosal immunization route appears to be relevant for protection against mucosal pathogens such as *Chlamydia* [27,28,61]. Determining the best mucosal route for immunization is important not only for eliciting the strongest protection but for ease of use, cost, safety, and societal acceptance. The i.n. route combined with i.m. immunization has been shown to induce mucosal IgA and stimulate immunity not only in local tissues but also in the respiratory and genital tracts [27,28,61]. For example, Ralli-Jain et al. [61] reported that vaccination with recombinant MOMP, combining mucosal and systemic routes, provides enhanced protection against a *C. muridarum* respiratory challenge when compared with protocols using only mucosal or systemic routes for immunization. Similar results were also seen with influenza [62].

## 5. Conclusions

We have shown that *C. muridarum* MOMP formulated in tNLPs, with a combination of a Th1 and a Th2 adjuvant that was delivered by mucosal and systemic routes, elicits humoral and cell-mediated immune responses that partially protect mice against a respiratory challenge. This approach may be used for the formulation of other membrane protein-based vaccines, as the tNLP platform may help promote the formation of native conformation of recombinantly expressed membrane proteins and enhance membrane protein solubility. tNLPs are also highly amenable to co-localized delivery of antigens and adjuvants.

## Data Availability

Data is contained within the article or supplementary material.

## Supplementary Materials

You can find supplementary materials in the attachment online at https://www.mdpi.com/article/10.3390/vaccines9070755/s1. Figure S1: Purification and characterization of MOMP-telodisk; Figure S2: Changes in body weight following the i.n. challenge; Figure S3: Changes in body weight, lungs weight and number of C. muridarum IFU recovered from the lungs a D10 following the i.n. challenge; Figure S4: Full figures of gel and western blot images.

## Abbreviations

Amphipols (Apols); antigen presenting cells (APC); Chlamydia (C.); constant domain (CD); day 1 post-challenge (D1 p.c.); dithiothreitol (DTT); elementary bodies (EB); enzyme linked immunosorbent assay (ELISA); geometric mean titer (GMT); inclusion forming units (IFU); intranasal (i.n.); intramuscular (i.m.); lymphoproliferative assay (LPA); major outer membrane protein (MOMP); telodendrimer nanolipoprotein particles (tNLP); size exclusion chromatography (SEC); sodium dodecyl sulfate-polyacrylamide gel electrophoresis (SDS-PAGE); variable domain (VD).
