# Supplementary material for: Induction of Protection in Mice against a Chlamydia muridarum Respiratory Challenge by a Vaccine Formulated with the Major Outer Membrane Protein in Nanolipoprotein Particles
Source: Vaccines (Basel). 2021 Jul 7;9(7):755. doi: 10.3390/vaccines9070755 (PMC8310061; doi:10.3390/vaccines9070755)
Supplement: Supplementary file 1 [file vaccines-09-00755-s001.zip › vaccines-1218111-supplementary.pdf]

Supplemental Material:

Suppl. Fig 1

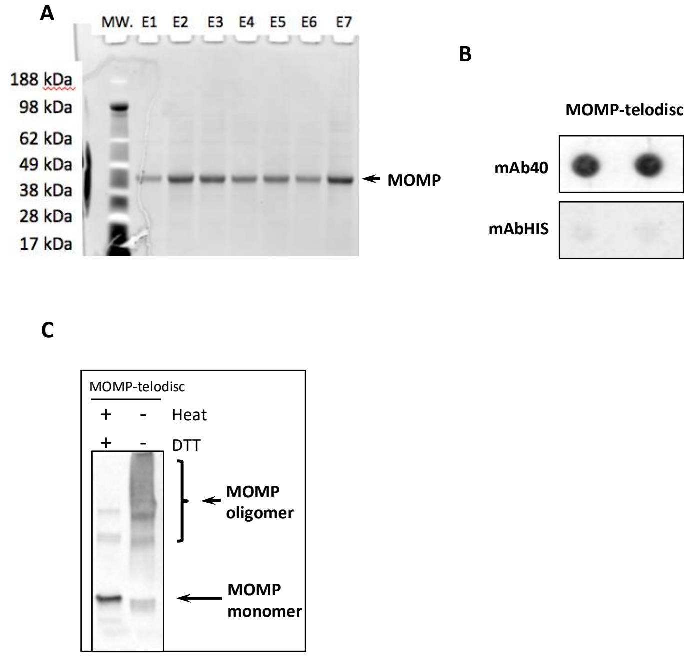

Figure S1: Purification and characterization of MOMP-telodisk (A) SDS-PAGE of Ni elutions of MOMP-telodisk. The gel densitometry values are: lane 1, 3412.4; lane 2, 7851.5; lane 3, 6709.9; lane 4, 5178.5; lane 5, 5346.8; lane 6, 4242.9; lane 7, 11107.4 (Analyzed by ImageJ). (B) Dot blot of purified MOMP-telodisk probed with anti-MOMP mAb40, and anti-Histag antibody mAbHIS. Blotting is done in duplicate. Histag is on the telodendrimer molecule, which may be less accessible to antibody binding and cause the anti-Histag signal to be significantly lower. (C) MOMP forms higher order structures in MOMP-telodisk as analyzed by Western blot. The gel densitometry values are: left lane top bands, 2141.4; left lane bottom band, 6393.3; right lane top bands 6343.9; right lane bottom band 2816.7 (Analyzed by ImageJ).

Suppl. Fig. 2

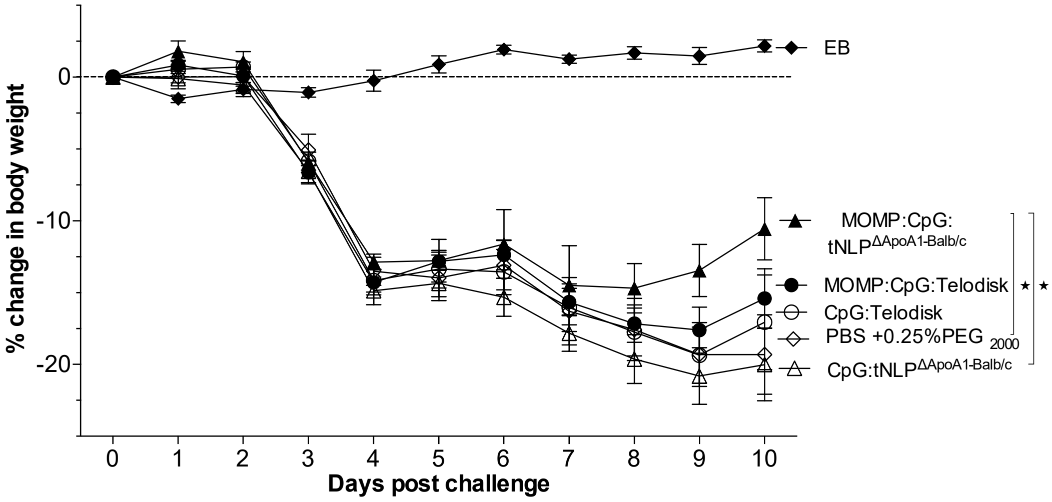

Figure S2. Changes in body weight following the i.n. challenge Following the i.n. challenge mice were weighed daily. Shown are the percentage changes in mean body weight +/−1SE) over the 10 experimental days.* $p < 0.05$ by the Repeated Measures 2-way ANOVA.

Suppl. Fig. 3

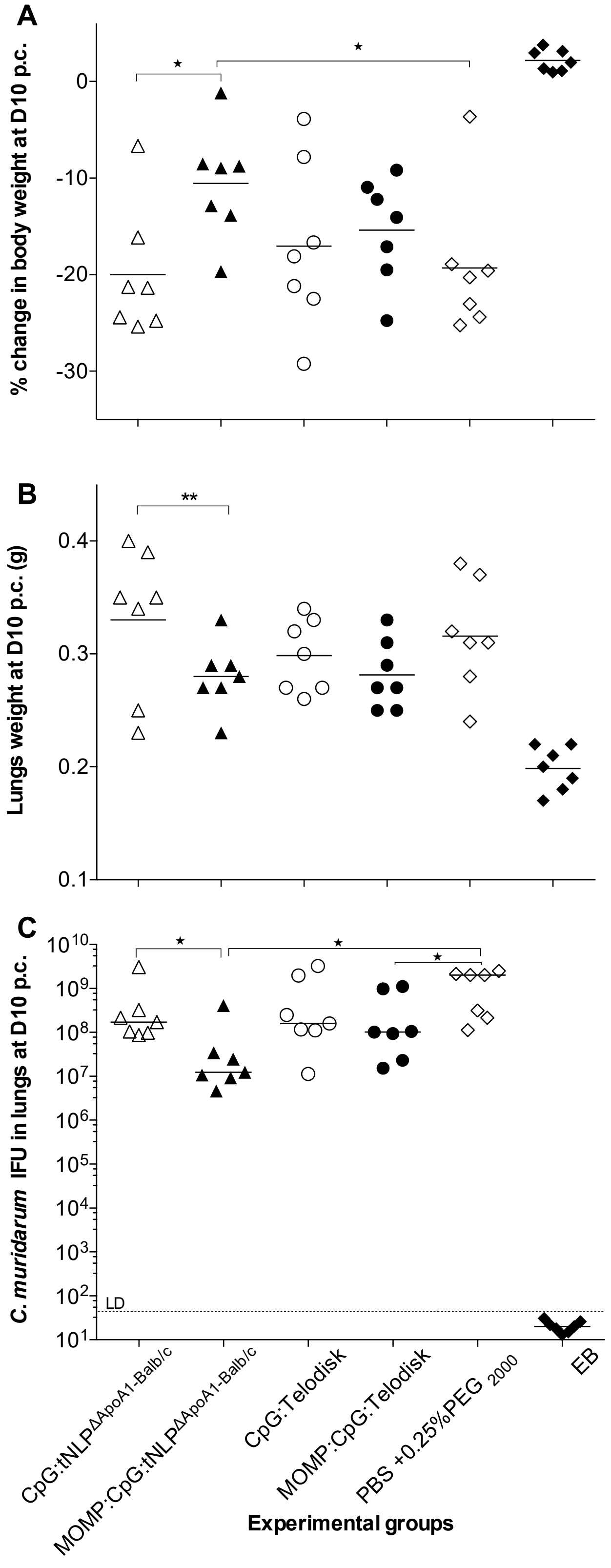

Figure S3. Changes in body weight, lungs weight and number of C. muridarum IFU recovered from the lungs a D10 following the i.n. challenge. (A) Percentage change in mean body weight at D10 following the i.n. challenge. The mean is shown as a horizontal line. Each symbol represents a single animal. (B) Lungs weight (g) at D10 after the i.n. challenge. The mean is shown as a horizontal

line. Each symbol represents a single animal. (C) Number of C. muridarum IFU recovered from the lungs at D10 after the i.n. challenge. The median is shown as a horizontal line. Each symbol represents a single animal.

Suppl. Fig 4

A

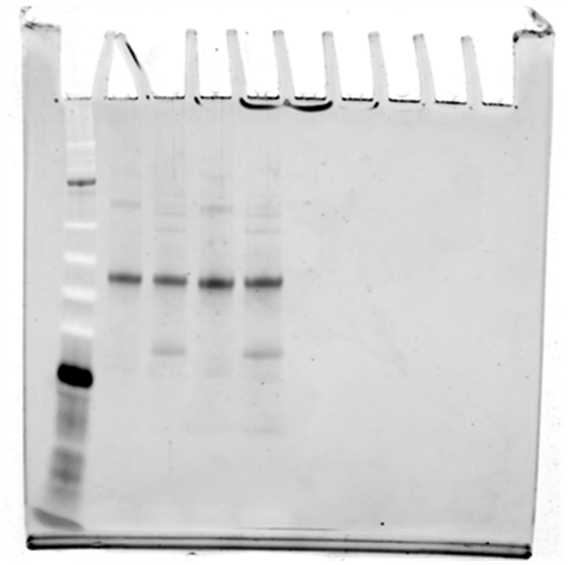

B

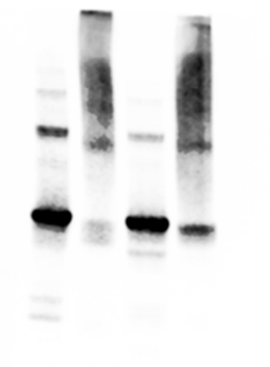

C

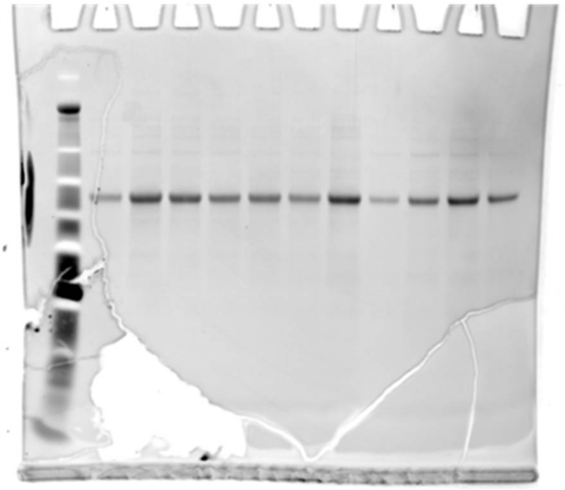

D

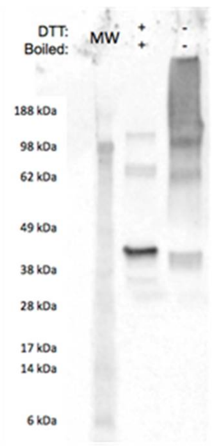

Figure S4. Full figures (A) Full figure of Figure 1A, SDS-PAGE of purified MOMP-tNLP. (B) Full figure of Figure 1C, MOMP forms higher order structures in MOMP-tNLPs as analyzed by Western blot. (C) Full figure of Figure S1A, SDS-PAGE of Ni elutions of MOMP-telodisk. (D) Full figure of Figure S1C, MOMP forms higher order structures in MOMP-telodisk as analyzed by Western blot.
